# Loss of males from mixed-sex societies in termites

**DOI:** 10.1186/s12915-018-0563-y

**Published:** 2018-09-25

**Authors:** Toshihisa Yashiro, Nathan Lo, Kazuya Kobayashi, Tomonari Nozaki, Taro Fuchikawa, Nobuaki Mizumoto, Yusuke Namba, Kenji Matsuura

**Affiliations:** 1 School of Life and Environmental Sciences, Edgeworth David Building A11, University of Sydney, Sydney, NSW 2006 Australia; 2 Laboratory of Insect Ecology, Graduate School of Agriculture, Kyoto University, Kyoto, 606-8502 Japan

**Keywords:** All-female asexual societies, Asexual social lineages, Thelytokous parthenogenesis, Sexual reproduction, Advanced social animals, Social insects

## Abstract

**Background:**

Sexual reproduction is the norm in almost all animal species, and in many advanced animal societies, both males and females participate in social activities. To date, the complete loss of males from advanced social animal lineages has been reported only in ants and honey bees (Hymenoptera), whose workers are always female and whose males display no helping behaviors even in normal sexual species. Asexuality has not previously been observed in colonies of another major group of social insects, the termites, where the ubiquitous presence of both male and female workers and soldiers indicate that males play a critical role beyond that of reproduction.

**Results:**

Here, we report asexual societies in a lineage of the termite *Glyptotermes nakajimai*. We investigated the composition of mature colonies from ten distinct populations in Japan, finding six asexual populations characterized by a lack of any males in the reproductive, soldier, and worker castes of their colonies, an absence of sperm in the spermathecae of their queens, and the development of unfertilized eggs at a level comparable to that for the development of fertilized eggs in sexual populations of this species. Phylogenetic analyses indicated a single evolutionary origin of the asexual populations, with divergence from sampled sexual populations occurring about 14 million years ago. Asexual colonies differ from sexual colonies in having a more uniform head size in their all-female soldier caste, and fewer soldiers in proportion to other individuals, suggesting increased defensive efficiencies arising from uniform soldier morphology. Such efficiencies may have contributed to the persistence and spread of the asexual lineage. Cooperative colony foundation by multiple queens, the single-site nesting life history common to both the asexual and sexual lineages, and the occasional development of eggs without fertilization even in the sexual lineage are traits likely to have been present in the ancestors of the asexual lineage that may have facilitated the transition to asexuality.

**Conclusions:**

Our findings demonstrate that completely asexual social lineages can evolve from mixed-sex termite societies, providing evidence that males are dispensable for the maintenance of advanced animal societies in which they previously played an active social role.

**Electronic supplementary material:**

The online version of this article (10.1186/s12915-018-0563-y) contains supplementary material, which is available to authorized users.

## Background

Sexual reproduction is nearly ubiquitous in nature [1] and is thought to provide key selective advantages [2]. Sexual reproduction enables gene pools to be constantly mixed and generates new combinations of genes, facilitating adaptation to changing environments [3]. Nevertheless, some organismal lineages can exist without sex [1, 4]. Although asexual lineages occur sporadically in diverse animal taxa [1, 4], their sexual ancestors are often thought to have been pre-adapted genetically and/or ecologically for persistence following the loss of sexual reproduction [5, 6].

In the context of social insect colonies, the genetic diversity resulting from sexual reproduction is thought to provide multiple benefits, including enhanced disease resistance and resilient division of labour [7]. Despite these benefits, sexual reproduction is bypassed in advanced societies of some hymenopteran insects (ants and honeybees), leading to completely asexual lineages [8, 9]. Indeed, hymenopteran social insect species are thought to be pre-adapted to thelytokous parthenogenesis (asexual production of diploid females), owing to their haplodiploid sex determination system, where males are produced via arrhenotokous parthenogenesis (asexual production of haploid males) [5, 9]. Furthermore, among hymenopteran social insects, including normal sexual species, workers are always female; males display no helping behavior (i.e., societies are essentially all-female). This is thought to be an outcome of sex-specific pre-adaptations for helping in the solitary ancestors of social species [10].

**Fig. 1:**
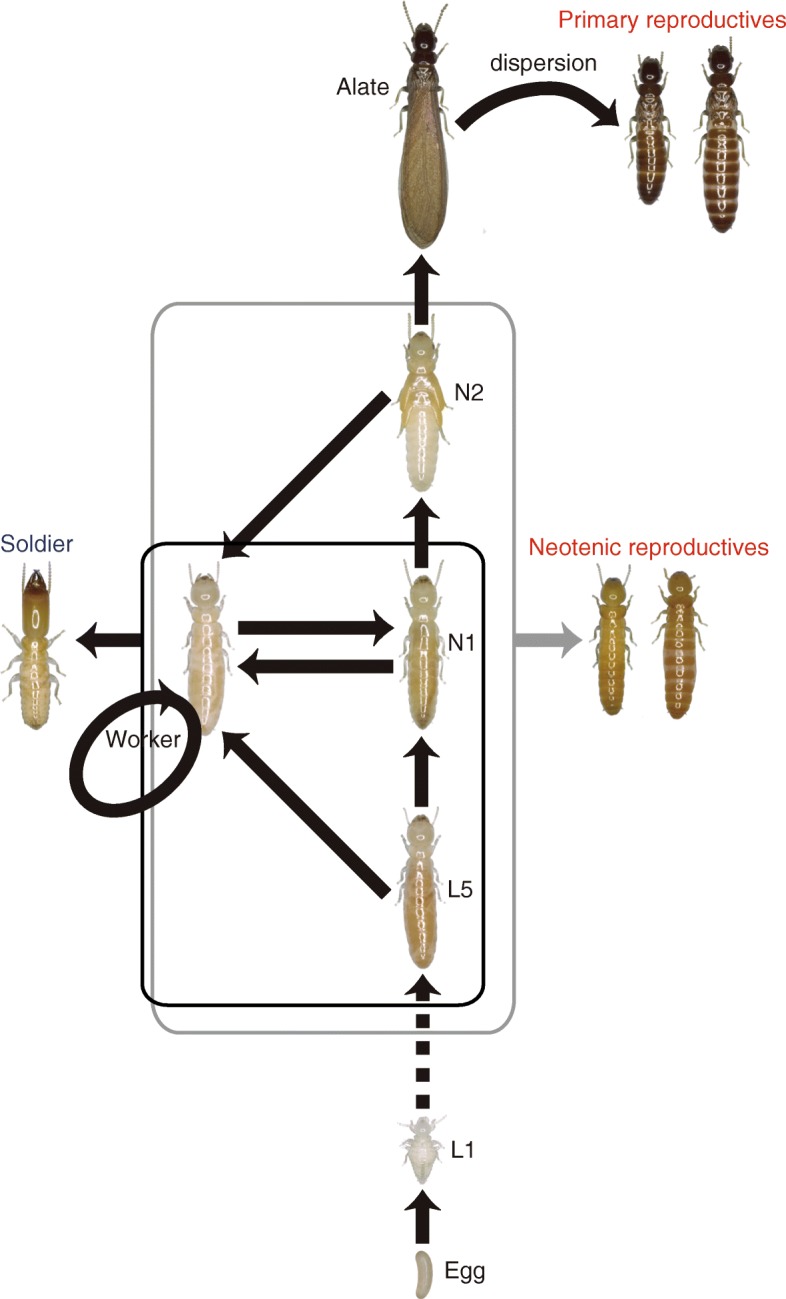
The caste-developmental pathway in Kalotermitidae. L1–L5, first- to fifth-instar larva; N1–N2, first- to second-instar nymph

In contrast to hymenopteran social insects, termites are diploid and typically have an XY sex determination system [11]. Their colonies commonly comprise both male and female reproductives, workers, and soldiers [12]. For example, although there is a variety of caste developmental pathways in termites, males and females of dry-wood termites (family Kalotermitidae) follow the same developmental pathway, through which the worker caste has totipotency to differentiate into other castes, including those of reproductives and soldiers [13, 14] (Fig. 1). The complete loss of males from termite lineages would therefore result not only in the loss of sexual reproduction, but also the loss of sex differences in the workforce (often due to size dimorphism) [15]. Termite parthenogenesis is expected to be thelytokous rather than arrhenotokous because of the XY sex determination system found in most species [16]. Although thelytokous parthenogenesis has been reported in some termites, this is restricted to the production of neotenic (secondary) reproductives in nature [16, 17] (but see also [18, 19] for the ability of parthenogenetic colony foundation of these “asexual queen succession (AQS)” termites in the laboratory). Importantly, both male and female workers and soldiers continue to be produced sexually in natural AQS colonies [16, 17], indicating that males play a critical role beyond that of reproduction in termite societies. To date, no termite colony in the field has ever been found to completely lack males.

The dry-wood termite *Glyptotermes nakajimai* Morimoto (Isoptera: Kalotermitidae) is widely distributed in coastal areas of the mainland (Honshu, Shikoku, and Kyushu) and small island regions (Amami-Oshima Island, Okinawa Islands, and Ogasawara Islands) of Japan [20, 21]. Previous studies of this species showed that their colonies contain both males and females [22]. However, our preliminary examinations indicated that colonies in some populations are exclusively female, including both worker and reproductive castes. We therefore hypothesized the evolutionary loss of males from representatives of this species.

We tested this hypothesis in a series of morphological, molecular, and cytological analyses, including sexing of individual termites and examination of sperm storage among egg-laying queens. Based on phylogenetic evidence of a single origin of asexuality, we further explored the evolution of the asexual lineage through comparative studies of asexual and sexual *G*. *nakajimai*.

## Results and discussion

### *Glyptotermesnakajimai* contains multiple asexual populations

**Fig. 2:**
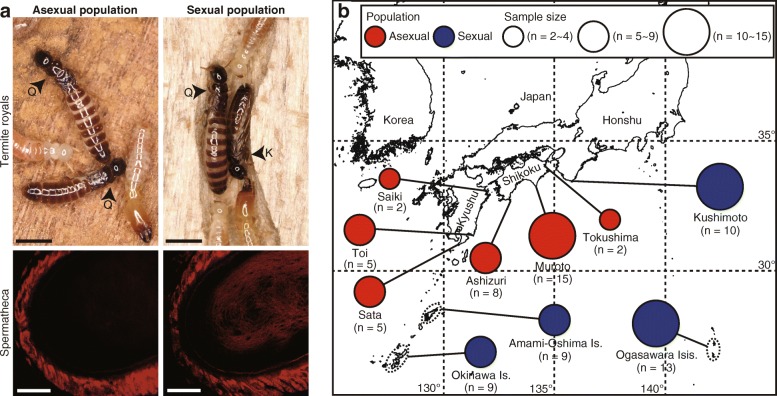
Asexual and sexual populations of the termite *Glyptotermes nakajimai*. **a** Termite royals in mature colonies of asexual (top left) and sexual (top right) populations, and spermathecae of egg-laying queens without sperm in an asexual population (bottom left) and with sperm in a sexual population (bottom right). Spermathecae were stained by propidium iodide and observed under a confocal fluorescence microscope. Q, queen; K, king. Scale bars, 2 mm (top); 20 μm (bottom). **b** Geographical distribution of asexual and sexual populations. Each population may include more than one collection site if they are located less than 50 km apart. The number of colonies sampled in each population is shown in parentheses

To confirm the presence of asexual populations of *G*. *nakajimai*, we investigated the colony composition of 74 mature colonies from ten populations. Colonies contained multiple reproductives and up to 2300 non-reproductive members (soldiers, workers, nymphs, alates, and young instars; see Fig. 1). In lower termites, including the family Kalotermitidae, individuals (except young instars) usually exhibit sexual dimorphism in their external morphology, such as the female-specific character of having an elongated seventh sternite [23, 24] (but see [25]). Dissections revealed that our sexing of *G*. *nakajimai* individuals on the basis of the configuration of the caudal sternites was 100% accurate in reproductives (*n* = 30), soldiers (*n* = 30), and workers (*n* = 60) (Additional file 1: Figure S1), confirming that the sex of these castes of *G*. *nakajimai* can be easily determined from sternite morphology. In the Shikoku and Kyushu populations, all reproductives (*n* = 194), soldiers (*n* = 316), and workers (*n* = 3700) collected from 37 colonies were female (Fig. 2a and Table 1). In contrast, all 37 colonies collected from the other populations (Honshu, Amami-Oshima Island, Okinawa Island, and Ogasawara Islands) contained both male and female reproductives (females/[females + males] = 0.53 ± 0.02 SEM [*n* = 548]), soldiers (females/[females + males] = 0.42 ± 0.03 SEM [*n* = 713]), and workers (females/[females + males] = 0.49 ± 0.01 SEM [*n* = 3700]) (Fig. 2a and Table 2).

**Table 1:** Compositions of mature field colonies in asexual populations

		No. reproductives (f/[f + m])			No. soldiers (f/[f + m])	No. workers (f/[f + m])
Colony code^a^	Location	A-derived	Neo.	Total		
	Shikoku, Japan					
TO150911A	Tokushima, Tokushima	6/6	1/1	7/7	17/17	100/100
TO150911B	Tokushima, Tokushima	2/2	1/1	3/3	4/4	100/100
MR150216B	Muroto, Kochi	1/1	1/1	2/2	9/9	100/100
MR150216C	Muroto, Kochi	1/1	1/1	2/2	3/3	100/100
MR150216E	Muroto, Kochi	2/2	0/0	2/2	8/8	100/100
MR150216G	Muroto, Kochi	5/5	0/0	5/5	14/14	100/100
MR150216H	Muroto, Kochi	3/3	1/1	4/4	11/11	100/100
MR150216I	Muroto, Kochi	6/6	0/0	6/6	15/15	100/100
MR150216J	Muroto, Kochi	5/5	1/1	6/6	12/12	100/100
MR150216K	Muroto, Kochi	3/3	2/2	5/5	12/12	100/100
MR150216M	Muroto, Kochi	2/2	0/0	2/2	8/8	100/100
MR150217B	Muroto, Kochi	5/5	5/5	10/10	17/17	100/100
MR150217C	Muroto, Kochi	2/2	1/1	3/3	4/4	100/100
MR150217D	Muroto, Kochi	2/2	2/2	4/4	13/13	100/100
MR150910A	Muroto, Kochi	12/12	1/1	13/13	14/14	100/100
MR150910B	Muroto, Kochi	7/7	1/1	8/8	22/22	100/100
MR150910D	Muroto, Kochi	16/16	0/0	16/16	13/13	100/100
AS141110A	Ashizuri, Kochi	10/10	1/1	11/11	17/17	100/100
AS141110B	Ashizuri, Kochi	5/5	0/0	5/5	1/1	100/100
AS141110D	Ashizuri, Kochi	6/6	3/3	9/9	6/6	100/100
AS141111A	Ashizuri, Kochi	3/3	2/2	5/5	1/1	100/100
AS141111H	Ashizuri, Kochi	3/3	0/0	3/3	6/6	100/100
AS141111I	Ashizuri, Kochi	2/2	0/0	2/2	17/17	100/100
AS141111J	Ashizuri, Kochi	2/2	0/0	2/2	1/1	100/100
AS141111K	Ashizuri, Kochi	8/8	2/2	10/10	16/16	100/100
	Kyushu, Japan					
SK150715A	Saiki, Oita	2/2	3/3	5/5	3/3	100/100
SK150715B	Saiki, Oita	2/2	0/0	2/2	1/1	100/100
TI150324A	Toi, Miyazaki	3/3	0/0	3/3	7/7	100/100
TI150324B	Toi, Miyazaki	2/2	0/0	2/2	1/1	100/100
TI150324C	Toi, Miyazaki	0/0	3/3	3/3	4/4	100/100
TI150324D	Toi, Miyazaki	4/4	0/0	4/4	3/3	100/100
TI150728A	Toi, Miyazaki	2/2	1/1	3/3	7/7	100/100
ST150322C	Sata, Kagoshima	2/2	0/0	2/2	7/7	100/100
ST150323L	Sata, Kagoshima	2/2	0/0	2/2	3/3	100/100
ST160304A	Sata, Kagoshima	4/4	0/0	4/4	4/4	100/100
ST160304B	Sata, Kagoshima	15/15	0/0	15/15	13/13	100/100
ST160304C	Sata, Kagoshima	4/4	0/0	4/4	2/2	100/100
Total		161/161	33/33	194/194	316/316	3700/3700
Mean (SEM)				100 (0)	100 (0)	100 (0)

All collected reproductives, soldiers, and 100 workers from each colony were sexed. A-derived: alate-derived; Neo: neotenic; f/[f + m], females/[females + males]

^a^Numbers in colony codes indicate the dates when colonies were collected. For example, colony TO150911A was collected on 11 September 2015

**Table 2:** Compositions of mature field colonies in sexual populations

		No. reproductives (f/[f + m])			No. soldiers (f/[f + m])	No. workers (f/[f + m])
Colony code^a^	Location	A-derived	Neo.	Total		
	Honshu, Japan					
IZ150430A	Izumo, Kushimoto, Wakayama	13/22	0/1	13/23	2/11	53/100
IZ150430B	Izumo, Kushimoto, Wakayama	3/5	0/1	3/6	2/7	49/100
IZ150430C	Izumo, Kushimoto, Wakayama	1/2	0/1	1/3	6/17	62/100
SN150430B	Misaki, Kushimoto, Wakayama	31/53	2/3	33/56	19/30	58/100
SN150430C	Misaki, Kushimoto, Wakayama	36/68	11/25	47/93	17/29	54/100
SN150430D	Misaki, Kushimoto, Wakayama	5/10	1/1	6/11	7/9	52/100
SN150430E	Misaki, Kushimoto, Wakayama	3/6	3/4	6/10	2/7	68/100
SN150430F	Misaki, Kushimoto, Wakayama	3/6	0/1	3/7	4/8	47/100
SN150430G	Misaki, Kushimoto, Wakayama	13/25	2/2	15/27	5/12	50/100
SN150501A	Misaki, Kushimoto, Wakayama	5/8	10/14	15/22	14/33	49/100
	Amami-Oshima Is., Japan					
NZ150526A	Naze, Kagoshima	3/9	3/3	6/12	2/8	44/100
NZ150526C	Naze, Kagoshima	1/2	3/4	4/6	4/12	60/100
NZ150526F	Naze, Kagoshima	8/19	0/0	8/19	6/15	55/100
NK150527A	Setouchi, Kagoshima	0/1	3/4	3/5	3/16	49/100
NK150527C	Setouchi, Kagoshima	3/6	0/0	3/6	13/22	53/100
	Okinawa Is., Japan					
HD150225A	Hedo, Okinawa	3/8	0/0	3/8	6/17	41/100
HD150225B	Hedo, Okinawa	4/7	7/14	11/21	5/37	40/100
HD150225C	Hedo, Okinawa	1/2	3/4	4/6	4/6	43/100
HD150225D	Hedo, Okinawa	2/3	1/1	3/4	3/8	42/100
HD160328B	Hedo, Okinawa	2/4	0/0	2/4	0/9	48/100
HD160328C	Hedo, Okinawa	2/4	0/0	2/4	5/15	59/100
NJ150226A	Nakijin, Okinawa	0/1	2/6	2/7	1/4	27/100
YJ150226A	Yagajijima, Okinawa	2/4	0/0	2/4	6/11	45/100
YJ150226B	Yagajijima, Okinawa	1/3	0/0	1/3	2/7	48/100
	Ogasawara Isis., Japan					
CC151014A	Chichijima, Tokyo	22/45	1/2	23/47	8/19	52/100
CC151014B	Chichijima, Tokyo	1/2	0/0	1/2	1/10	51/100
CC151014G	Chichijima, Tokyo	0/1	5/5	5/6	7/13	42/100
CC151014H	Chichijima, Tokyo	3/5	0/0	3/5	10/19	43/100
CC151015A	Chichijima, Tokyo	1/3	2/3	3/6	14/21	43/100
CC151015B	Chichijima, Tokyo	7/14	6/10	13/24	17/36	38/100
CC151015C	Chichijima, Tokyo	2/4	0/0	2/4	12/20	53/100
CC151015H	Chichijima, Tokyo	1/3	0/0	1/3	10/14	53/100
HH151016A	Hahajima, Tokyo	0/0	2/3	2/3	8/13	47/100
HH151016D	Hahajima, Tokyo	6/10	0/0	6/10	38/83	56/100
HH151016E	Hahajima, Tokyo	12/23	0/0	12/23	4/37	24/100
HH151016F	Hahajima, Tokyo	11/25	0/0	11/25	29/44	46/100
HH151016H	Hahajima, Tokyo	11/21	1/2	12/23	12/34	60/100
Total		222/434	68/114	290/548	308/713	1804/3700
Mean (SEM)				0.53 (0.02)	0.42 (0.03)	0.49 (0.01)

All collected reproductives, soldiers, and 100 workers from each colony were sexed. A-derived, alate-derived; Neo., neotenic; f/[f + m], females/[females + males]

^a^Numbers in colony codes indicate the dates when colonies were collected. For example, colony IZ150430A was collected on 30 April 2015

To rule out the possibility that cryptic males exist in the all-female populations (Shikoku and Kyushu), we examined sperm storage among egg-laying queens by observing the spermathecae (sperm storage organs) stained by propidium iodide. Spermathecae of all examined queens (*n* = 12) in the all-female populations of *G*. *nakajimai* were empty, whereas those of all examined queens (*n* = 12) in the other mixed-sex populations were filled with sperm (*P* < 0.0001, Fisher’s exact probability test) (Fig. 2a). This is, to our knowledge, the first demonstration of queens in natural colonies that lack sperm in their spermathecae. In all the cases, we could find in the literature every termite queen in natural colonies that has been checked has had sperm in her spermatheca, regardless of the presence or absence of the kings (e.g., [26, 27]).

**Fig. 3:**
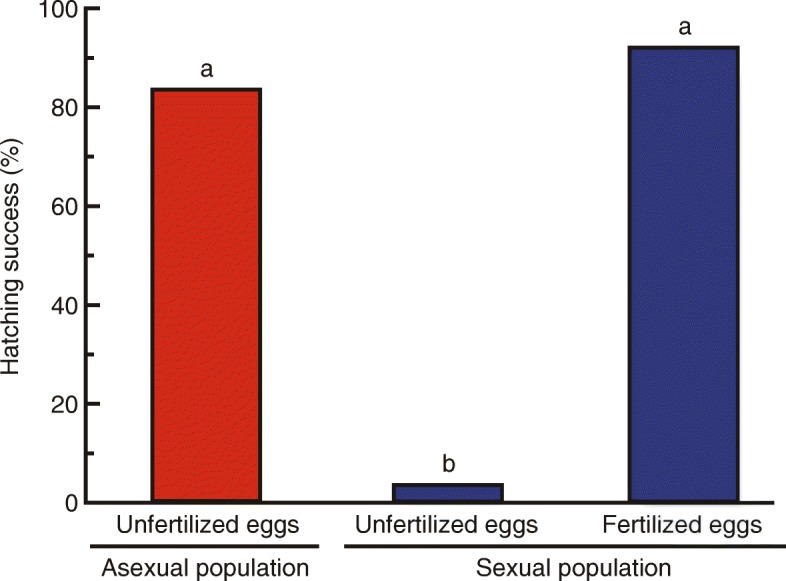
Increased hatching success of unfertilized eggs in asexual populations. Comparison of the percentage of eggs hatched within 100 days after colony foundation among unfertilized eggs of an asexual population (*n* = 134), unfertilized eggs of a sexual population (*n* = 193), and fertilized eggs of a sexual population (*n* = 127). Different letters on the bars indicate significant differences (*P* < 0.0001, Fisher’s exact probability test with Bonferroni correction). For raw data, see Additional file 7

We also compared the hatching success of unfertilized eggs between all-female and mixed-sex populations. As expected, the all-female populations exhibited high rates of hatching success of unfertilized eggs (112 of 134 eggs [83.6%] hatched) (Fig. 3). Notably, some unfertilized eggs from the mixed-sex population also hatched (7 of 193 eggs [3.6%] hatched), indicating the presence of tychoparthenogenesis (occasional development of eggs without fertilization) [1, 28, 29] in sexual colonies of this species (Fig. 3). No significant difference in hatching success was observed between unfertilized eggs of the all-female population (112 of 134 eggs [83.6%] hatched) and fertilized eggs of the mixed-sex population (117 of 127 eggs [92.1%] hatched) (*P* = 0.12, Fisher’s exact probability test with Bonferroni correction) (Fig. 3). Overall, our results clearly indicate complete asexuality in the Shikoku and Kyushu populations, in contrast to the other normal sexual populations (Fig. 2b).

### A single origin of asexuality in *G*. *nakajimai*

**Fig. 4:**
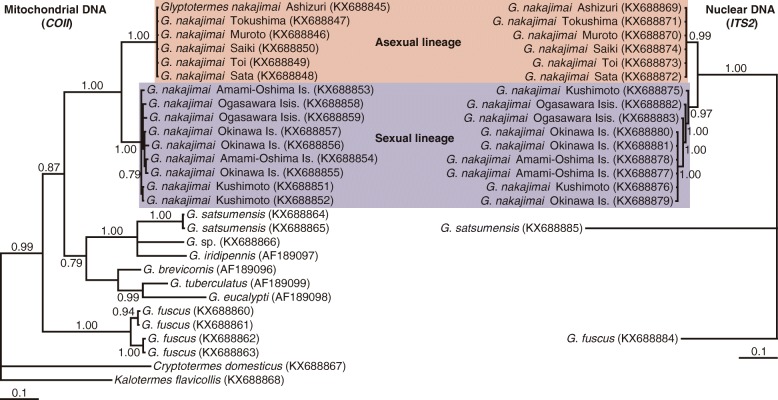
Evolutionary relationships among asexual and sexual populations of *Glyptotermes nakajimai*. Phylogenetic trees were obtained by Bayesian analyses of mitochondrial *COII* (left) and nuclear *ITS2* (right) sequences of *G*. *nakajimai* individuals representing each of the collection sites. The asexual lineage is highlighted in red, and the sexual lineage is highlighted in blue. Posterior probabilities (≥ 0.70) are shown at each node. The horizontal bar represents a distance of 0.1 substitutions per site. Multiple *Glyptotermes* spp. as well as *Cryptotermes domesticus* and *Kalotermes flavicollis* were used as outgroups. GenBank accession numbers are shown in parentheses. The topologies shown were very similar to those derived from maximum likelihood analyses, with some minor differences (see Additional file 2: Figure S2)

**Fig. 5:**
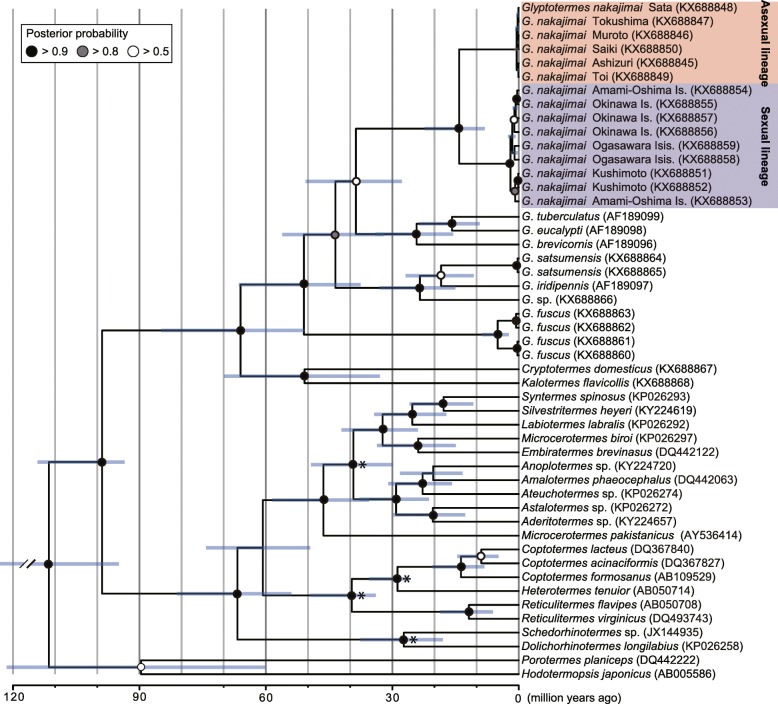
Chronogram showing divergence times among Kalotermitidae, including the asexual and sexual lineages of *Glyptotermes nakajimai*. The tree was inferred based on an alignment of mitochondrial *COII* sequences, using the program BEAST v1.8.2. The asexual lineage is highlighted in red, and the sexual lineage is highlighted in blue. Branch lengths are drawn to a time scale given in millions of years. Bars represent 95% confidence intervals for estimates of node times. Four fossil termites provided minimum age constraints for calibration of the molecular clock at the nodes denoted by asterisks (see “Methods” for further details concerning these fossils)

To elucidate the evolutionary relationships among asexual and sexual populations of *G*. *nakajimai*, we conducted phylogenetic analyses of *Glyptotermes* termites based on two independently evolving markers: mitochondrial cytochrome *c* oxidase subunit II (*COII*) and nuclear internal transcribed spacer 2 (*ITS2*) sequences. The monophyly of *G*. *nakajimai* was unequivocally supported in each analysis (*COII*: Bayesian posterior probability [BPP] = 1.00; RAxML bootstrap support [RBS] = 100%, *ITS2*: BPP = 1.00; RBS = 100%) (Fig. 4, Additional file 2: Figure S2). All sequences of *COII* and *ITS2* from six collection sites across the range of asexual populations were identical (*COII*: 100% identity, *ITS2*: 100% identity). Likewise, *COII* and *ITS2* sequences from nine collection sites across the range of sexual populations were highly similar to each other (*COII*: 99%–100% identity, *ITS2*: 96%–100% identity), and the monophyly of all sexual populations was well-supported (*COII*: BPP = 1.00; RBS = 93%, *ITS2*: BPP = 0.97; RBS = 100%) (Fig. 4, Additional file 2: Figure S2). Given that all other known species of termites, including members of the genus *Glyptotermes*, are sexual, these results demonstrate a single origin of asexuality within *G*. *nakajimai*. The estimated divergence time between the two lineages was 14.1 million years ago (95% confidence interval = 8.1–22.4 million years ago [Mya]) (Fig. 5). Whether asexuality has persisted in *G. nakajimai* for this period of time is not known. It is possible that the most closely related sexual relatives of the asexual *G. nakajimai* were not represented among our samples (perhaps because they are extinct, or found in areas we did not collect from), and that asexuality evolved much more recently than 14.1 Mya. Further work is required to investigate this issue.

**Fig. 6:**
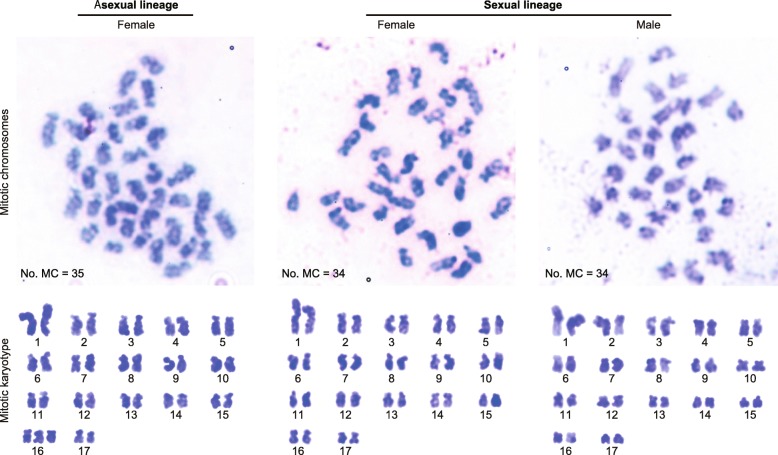
Proposed karyotype of *Glyptotermes nakajimai*. Top: mitotic chromosomes of a female of the asexual lineage (left), a female of the sexual lineage (middle), and a male of the sexual lineage (right). MC, Mitotic chromosome. Bottom: mitotic karyotypes of a female of the asexual lineage (2n = 35) (left), a female of the sexual lineage (2n = 34) (middle), and a male of the sexual lineage (2n = 34) (right)

Previous work has shown that the external morphology and cuticular hydrocarbon profiles of *G. nakajimai* representatives across its range are indistinguishable [21], including the asexual populations reported here. To further investigate potential fine-scale differences between the asexual and sexual lineages, we performed karyotyping. The sexual lineage consistently displayed 2n = 34 chromosomes (female: *n* = 12, male: *n* = 12), while the asexual lineage consistently harbored a complement of 2n = 35 (*n* = 18) and contained a trisomy, most probably of chromosome 16 (Fig. 6). To confirm our observations that the asexual lineage has an extra chromosome, we compared genome size (based on the *C* value or nuclear DNA mass) between the asexual and sexual lineages. As expected, the genome size of the asexual lineage was significantly higher than that of the sexual lineage (colony: *F*_4, 24_ = 0.80, *P* = 0.54; lineage: *F*_1, 24_ = 55.76, *P* < 0.0001; nested ANOVA with colonies nested within lineages [the asexual lineage and the sexual lineage]), although the ploidy levels were similar between the two lineages (Table 3). These results are consistent with the two lineages containing different chromosome profiles (Fig. 6), in addition to their molecular sequence profiles (Fig. 4, Additional file 2: Figure S2).

**Table 3:** Comparison of genome size (*C* value) between the asexual and sexual lineages

Lineage	*C*-value (pg)^a^	Relative ploidy level
Asexual lineage	1.27 ± 0.00****	2.07
Sexual lineage	1.23 ± 0.00	2

Data for the asexual lineage was compared with the sexual lineage using nested ANOVA. ****, *P* < 0.0001

^a^Values are mean ± SEM (*n* = 15)

### Greater uniformity of head size in all-female soldiers of asexual colonies

**Fig. 7:**
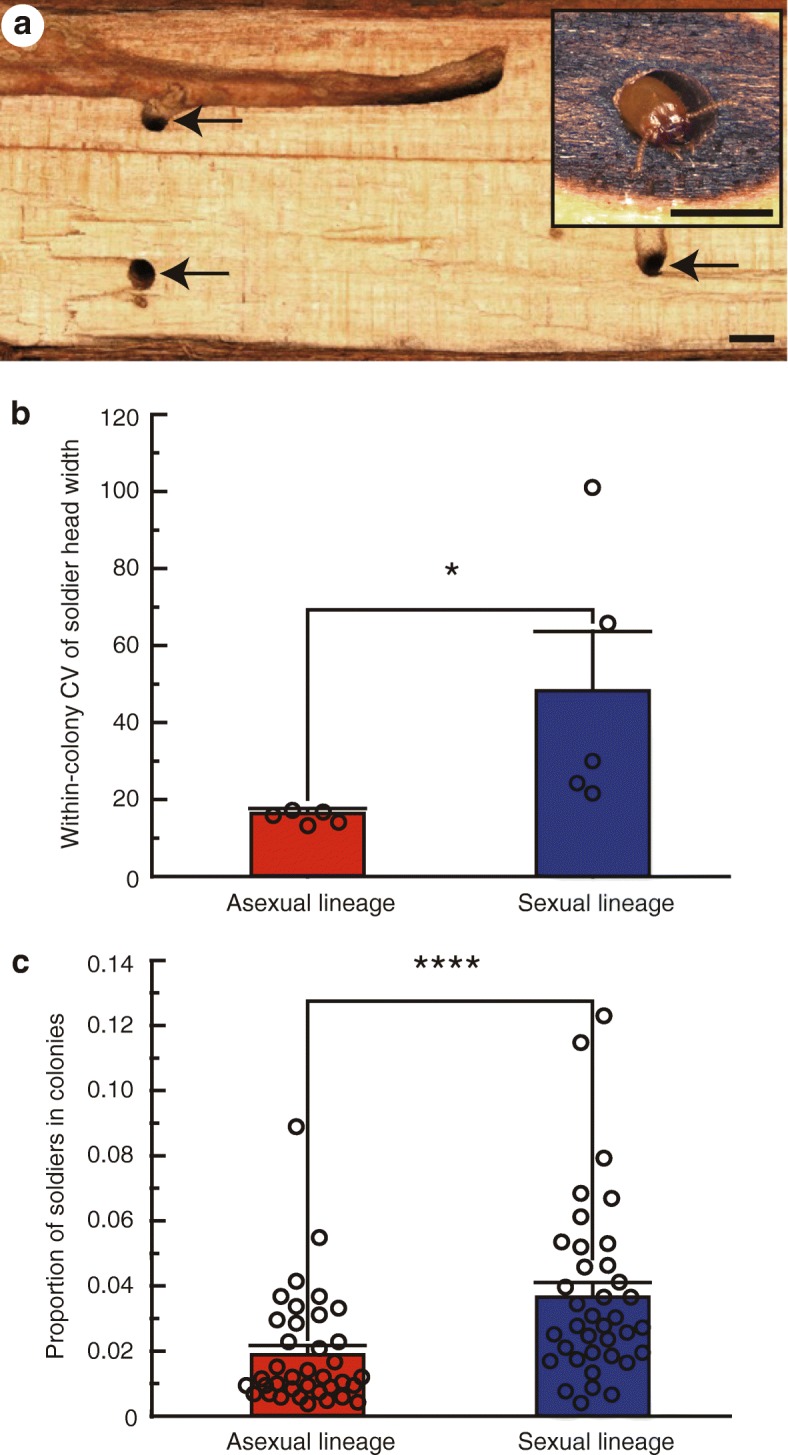
Defensive advantage of colonies in the asexual lineage. **a** Cross section showing the structure of a *Glyptotermes nakajimai* nest. Small tunnels connecting chambers are indicated by arrows. (inset, right) A tunnel-blocking soldier (phragmotic defense). Scale bars, 2 mm. **b** Comparison of the within-colony coefficient of variation (CV) of soldier head width between the asexual and sexual lineages. Values are mean ± SEM (*n* = 5). Individual data points are represented by open circles. *, *P* < 0.05 (Mann–Whitney *U* test). For raw data, see Additional file 7. **c** Comparison of the proportion of soldiers to other individuals of mature field colonies between the asexual and sexual lineages. Values are mean ± SEM (*n* = 37). Individual data points are represented by open circles. ****, *P* < 0.0001 (GLM). For raw data, see Additional file 7

Phenotypic variations among individuals within a colony often provide opportunities for efficient task partitioning, such as sexual specialization in tasks in animal societies [13, 15, 30, 31]. However, such variations among individuals performing the same task may lower task-efficiency when individual variation reduces group performance. The most common defense mechanism in lower termites, including the genus *Glyptotermes*, is phragmosis, where soldiers with plug-like heads block tunnels connecting chambers, thus preventing enemies from invading the nest [32, 33]. Tunnel width within a nest is relatively uniform, and stabilizing selection acts on soldier head width for efficient phragmotic defense in these termites, because soldiers with narrower head widths would tend to allow enemies to pass through more easily, while those with greater head width would tend to clog tunnels within the nest [34, 35]. When sampling *G. nakajimai* in the field, we found that individuals of several ant species carried off struggling workers when logs infested with *G*. *nakajimai* were broken open. However, soldiers typically retreated to the small tunnels between chambers and plugged the tunnels with their heads to prevent the ants from invading intact parts of the nest (i.e., phragmotic defense) (Fig. 7a).

In the sexual lineage of *G*. *nakajimai*, male soldiers were significantly smaller than female soldiers in head width (colony: *F*_4,191_ = 5.16, *P* < 0.001, sex: *F*_1,191_ = 6.86, *P* < 0.01, two-way ANOVA), as well as in head length (colony: *F*_4,191_ = 10.67, *P* < 0.0001, sex: *F*_1,191_ = 13.21, *P* < 0.001, two-way ANOVA) (Additional file 3: Figure S3). Stabilizing selection appears to act on soldier head width because the head width to length ratio differed significantly between the sexes (colony: *F*_4,191_ = 12.46, *P* < 0.0001, sex: *F*_1,191_ = 12.15, *P* < 0.001, two-way ANOVA), such that differences in head width between the sexes were reduced (Additional file 3: Figure S3).

To investigate whether asexuality leads to increased stabilization of soldier head width, we compared the within-colony coefficient of variation (CV) of soldier head width between the asexual and sexual lineages. The asexual lineage had significantly smaller within-colony CVs of soldier head width than those of the sexual lineage (*P* = 0.012, Mann–Whitney *U* test) (Fig. 7b), indicating reduced intracolonial variation in soldier head width in the asexual lineage. This is presumably due to a loss in sexual dimorphism in the asexual lineage, although the loss of genetic diversity resulting from parthenogenesis may also contribute. We also compared the proportion of soldiers to other individuals in mature field colonies between the asexual and sexual lineages. Mature field colonies of the asexual lineage had significantly lower proportions of soldiers to other individuals than those of the sexual lineage (*P* < 0.0001, GLM) (Fig. 7c), suggesting the number of soldiers required for colony defense in the asexual lineage appears to be lower than that in the sexual lineage. A reduction in the number of soldiers, which require sibling care because they are unable to feed themselves, is likely to allow investment elsewhere in the colony. Thus, our results hint at the possibility of increased defensive efficiencies arising from the greater morphological uniformity of soldiers in all-female asexual colonies. It is possible that such efficiencies contributed to the persistence and spread of the asexual lineage.

If the sexual lineage produced only female soldiers (or only male soldiers), intracolonial variation in soldier head width could also be reduced. Indeed, sexual specialization of soldiers is common in many termite taxa with sterile workers [13, 15]. However, such sexual specialization is extremely rare in dry-wood termites (Kalotermitidae) [13, 15]. This can be partly explained by their caste-developmental pathway, through which all colony members finally develop into alates, neotenic reproductives (only a small proportion of individuals), or sterile soldiers [13, 14] (Fig. 1). Sexual specialization of soldiers would therefore lead to skewed sex ratios of other colony members within colonies, most importantly alates. This would potentially lead to a failure of some alates from the colony to successfully breed.

### Pre-adaptive conditions for the evolution of maleless societies

When the costs of asexuality are not high, a mutation causing asexual reproduction is expected to rapidly spread within a sexual population due to the twofold reproductive advantage of asexual reproduction. Under this scenario, asexual females produce only female offspring, while sexual females continue to produce both male and female offspring [36]. Assuming that approximately equal numbers of offspring are produced by both asexual and sexual colonies, asexual females will increase in frequency in the population and eventually replace sexual females entirely unless mutations that suppress asexuality arise.

We hypothesize the existence of a number of key traits in the ancestors of asexual *G*. *nakajimai*, which permitted them to overcome the barriers required for complete loss of males from their mixed-sex societies. Firstly, we hypothesize that the sexual ancestors of asexual *G*. *nakajimai* were likely to have been pre-adapted to overcome at least some developmental constraints associated with parthenogenesis. This is because parthenogenetic eggs have to be activated without sperm, and centrioles need to be inherited only from mothers during reproduction [5]. This hypothesis is supported by our finding of tychoparthenogenesis (occasional development of eggs without fertilization) in the sexual lineage, whereby 7 of 193 unfertilized eggs (3.6%) successfully developed into larvae (Fig. 3). Tychoparthenogenesis is thought to provide an important pathway to the evolution of parthenogenesis [29], but is also relatively common in many animal groups, regardless of the presence or absence of asexual lineages [1, 28]. Therefore, tychoparthenogenesis is unlikely to be the only characteristic of the sexual ancestors of *G*. *nakajimai* that facilitated the evolution of all-female colonies.

The second trait we propose to have facilitated the transition to asexuality in *G*. *nakajimai* is cooperative colony foundation by queens (i.e., pleometrosis). Following the departure of alates from termite colonies on their nuptial flight, most termite incipient colonies are founded by a monogamous pair of primary (alate-derived) reproductives (i.e., a king and a queen) [37]. Both asexual and sexual *G. nakajimai* lineages produce alates that leave their colonies to undertake nuptial flights [21, 22]. However, rather than finding a single queen in incipient field colonies, we found that one incipient field colony of the asexual *G. nakajimai* lineage contained two queens, and three other incipient asexual field colonies were founded by more than two queens (range = 4–25). We found that the number of larvae per colony was positively correlated with the number of founders (Table 4). Incipient termite colonies typically experience high mortality rates, owing to disease and other factors [38, 39]. Therefore, increased numbers of colony members through cooperative colony foundation by reproductives would be adaptive, especially in asexual populations, given the presumed genetic disadvantages of parthenogenesis [40]. In the present study, we were not able to find incipient field colonies of the sexual lineage of *G*. *nakajimai*. However, we found that 29 of 37 mature field colonies (78.4%) of the sexual lineage, as well as 21 of 37 mature field colonies (56.8%) of the asexual lineage, contained more than two alate-derived reproductives (Tables 1 and 2). This is suggestive of cooperative colony foundation by multiple kings and queens, although we cannot rule out the possibility of colony fusion. Based on these results, we hypothesize that the ancestors of the asexual lineage benefited from multi-queen colony foundation during the transition to male-free societies.

**Table 4:** Compositions of incipient field colonies of the asexual lineage

Colony code^a^	Location	No. primary queens	No. larvae
MR150217A	Muroto, Kochi, Shikoku	25	51
AS141111B	Ashizuri, Kochi, Shikoku	2	8
AS141111D	Ashizuri, Kochi, Shikoku	6	17
ST150323F	Sata, Kagoshima, Kyushu	4	12
Mean (SEM)		9.25 (5.31)	22.00 (9.84)

^a^Numbers in colony codes indicate the dates when colonies were collected. For example, colony MR150217A was collected on 17 February 2015

The presumed presence of multi-queen colony foundation in the ancestor of asexual *G. nakajimai* is also likely to have removed one potential barrier to the spread of the asexual phenotype: the expected failure of many colonies founded by single asexual queens [41]. Termites cannot clean their own bodies by self-grooming, with the exception of their antennae. Mutual grooming by reproductives during incipient colony foundation is therefore thought to play a key role in disease avoidance [41]. During the evolution of termite all-female asexual societies, females that founded colonies with other females (rather than alone) are likely to have been at a significant advantage, both from the benefits of grooming, as well as faster colony growth due to enhanced reproductive output.

The final traits we hypothesize that may have permitted the loss of males in *G*. *nakajimai* are those generally associated with the life histories of dry-wood termites. Dry-wood termites are single-site nesters, living in a single piece of dead wood that serves both as nest and as food [42]. This means that individual members do not need to forage outside the nest, reducing the risk of exposure to pathogens and parasites. Importantly, dry-wood nesters can easily disperse over water through wood-rafting. This is evidenced by the presence of many species, including *G*. *nakajimai*, in coastal areas and on remote islands [20, 21, 43, 44]. According to the Red Queen hypothesis, sexual reproduction is favored because it helps the population to co-evolve with specialist parasites and pathogens [45]. Long-distance dispersal events might have released sexually reproducing ancestors of asexual *G*. *nakajimai* from selection pressures favoring sexual reproduction. This is because expansion into new areas may allow founders to escape from parasites and pathogens [46, 47]. Moreover, following dispersal to new habitats, asexuality could be adaptive because it is an effective way of circumventing the challenges associated with low population densities, such as inbreeding depression and the inability to find mates [48]. Indeed, many asexual lineages of ants are unusually widespread geographically [9].

### The mode of parthenogenesis in *G*. *nakajimai*

The transition from sexual to parthenogenetic reproduction may have significant negative consequences as a result of increased homozygosity and inbreeding depression [5]. Such increases in homozygosity may occur under two forms of automictic parthenogenesis: gamete duplication or terminal fusion of gametes produced during meiosis. On the other hand, heterozygosity may be maintained in offspring under alternative forms of automictic parthenogenesis: central fusion of gametes produced during meiosis, or apomixis (in which eggs cells are essentially clones produced via mitosis) [5]. Indeed, the mode of thelytokous parthenogenesis in asexual lineages of hymenopteran social insects is either automixis with central fusion or apomixis [8, 9]. The presence of an extra chromosome in all examined members of the *G*. *nakajimai* asexual lineage (2n = 35, vs 2n = 34 in members of the sexual lineage; Fig. 6) is suggestive of apomixis, as opposed to automixis. This is because an uneven number of chromosomes is likely to lead to pairing problems during the first stage of meiosis. Apomixis, on the other hand, would lead to a consistent number of chromosomes being passed to offspring [5]. We performed a preliminary investigation of the mode of parthenogenesis in *G*. *nakajimai* by genotyping individuals from both the asexual and sexual lineages (including offspring produced through tychoparthenogenesis from the sexual lineage; Fig. 3) at six polymorphic microsatellite markers developed for *Glyptotermes* termites (Additional file 4: Table S1). In the sexual lineage of *G*. *nakajimai*, offspring produced by tychoparthenogenesis were homozygous for a single maternal allele at two polymorphic loci (*Gly08* and *Gly18*; Additional file 5: Table S2) (Additional file 6: Table S3). This pattern is suggestive of either (a) automixis with terminal fusion, where offspring are homozygous for a single maternal allele at all loci if no crossing-over takes place, as reported in other lower termites [16, 49], or (b) automixis with gamete duplication where offspring are completely homozygous for a single maternal allele at all loci, as suggested in a study of higher termites [16]. Unfortunately, the six markers we analyzed showed no polymorphism in the asexual lineage of *G*. *nakajimai* (Additional file 5: Table S2). Therefore, further work involving deeper sampling of the genomes of *G*. *nakajimai* representatives is required to test the hypothesis that the mode of parthenogenesis in the asexual lineage is apomixis, as suggested by its uneven number of chromosomes (Fig. 6). If our hypothesis is correct, such sampling of the asexual and sexual lineages could also determine whether heterozygous genotypes in the asexual lineage (if present) are derived from the conservation of initial allelic diversity present in the sexual ancestor. Under this scenario, the asexual lineage would have evolved through a transition from normal sexual reproduction directly to apomixis, as is known in *Timema* stick insects [6]. Alternatively, any heterozygous genotypes in the asexual lineage could be derived from new mutations, indicating that the asexual lineage evolved through an automictic step (as appears to be the case in tychoparthenogenetic *G*. *nakajimai* (Additional file 6: Table S3)).

## Conclusion

Asexual lineages of advanced social animals were previously recognized only among Hymenoptera, whose societies are essentially all-female [8, 9]. In contrast to hymenopteran societies, the common presence in termite societies of both male and female workers and soldiers [12] indicates that a mixed-sex workforce is a key component of their life history. We have conclusively demonstrated the completely asexual nature of some *G*. *nakajimai* populations (Fig. 2a, b, Tables 1 and 2), and have shown that this evolved on a single occasion from sexual ancestors with mixed-sex colonies (Fig. 4, Additional file 2: Figure S2). Our findings show that, at least under certain ecological conditions, males are not essential for the maintenance of advanced animal societies in which they previously played an active social role.

We have shown that asexuality acts as a stabilizer of soldier head size, which we hypothesize is beneficial for efficient phragmotic defense in termites (Fig. 7b, c). We also propose that a combination of traits present in the ancestors of the asexual lineage, including tychoparthenogenesis (Fig. 3), colony foundation by multiple queens (Table 4), and a single-site nesting life history, facilitated the complete loss of males from mixed-sex societies. These traits presumably allowed a mutation causing asexuality to spread rapidly to fixation following its appearance in ancestral sexual populations, as expected due to the twofold reproductive advantage of asexual reproduction [36]. Although rigorous studies of these traits in termites are scarce, they might also apply to other termite species, especially in members of *Glyptotermes.* The presence of several primary reproductives within a colony, which is unusual for termites, seems to be relatively common in this genus (e.g., [50]). One reason why asexual termite lineages have not previously been discovered may be a lack of attention given to the sex of individual colony members, including workers and soldiers, which in termites are often collectively called “neuter castes” regardless of sex. The discovery of additional asexual lineages of termites in nature will be aided by morphological sexing of both reproductives and non-reproductives. Development of sex-linked genetic markers in many genera may allow for more convenient detection of asexuality in termites.

## Methods

### Termite collection

We collected 74 mature colonies and four incipient colonies, which comprise only primary reproductives and young larvae, of the dry-wood termite *G. nakajimai* from 15 sites across ten populations (Honshu [Kushimoto], Shikoku [Tokushima, Muroto, and Ashizuri], Kyushu [Saiki, Toi, and Sata], Amami-Oshima Island, Okinawa Island, and Ogasawara Islands, Japan) from November 2014 to March 2016 (Tables 1, 2, and 4). We very carefully dismantled nest woods and extracted all colony members using an aspirator and forceps. The reproductives (kings and queens), soldiers, workers (also called pseudergates), nymphs, alates, and young instars (see Fig. 1) from each colony were placed in a moist unwoven cloth in a 90-mm Petri dish and preserved at − 25 °C until further use. Portions of workers and nymphs from each colony were kept in the laboratory as stock colonies in 90-mm Petri dishes that contained damp chips of sliced Oregon pine wood at 25 °C under constant darkness until subsequent experiments. The sex of all collected reproductives, soldiers, and 100 workers from each colony was determined based on the configuration of the caudal sternites [23, 24] under a stereomicroscope (SZX7; Olympus, Tokyo, Japan). To compare the proportion of soldiers to other individuals of mature field colonies between asexual and sexual populations, we used general linear modeling (GLM) (JMP 9; SAS Institute, Cary, NC).

### Sexing *G. nakajimai* reproductives, soldiers, and workers

To test the accuracy of sexing individuals using external morphology in *G*. *nakajimai*, we used reproductives, soldiers, and workers of three mature field colonies of the sexual lineage (SN150430B, NZ150526F, and HH151016F in Table 2). Under a stereomicroscope (SZX7; Olympus, Tokyo, Japan), five putative male and five putative female reproductives, five putative male and five putative female soldiers, and ten putative male and ten putative female workers were randomly chosen from each colony using the configuration of the caudal sternites [23, 24]. The true sex was then ascertained through inspection of the gonads [51].

### Spermatheca analysis

To examine the insemination status of egg-laying queens, we developed a spermatheca staining method to observe inseminated sperm. Two egg-laying queens were randomly chosen from each of the six field colonies collected from asexual populations (TO150911A, TO150911B, MR150910A, MR150910B, SK150715A, and TI150728A in Table 1) and from sexual populations (IZ150430B, SN150430C, SN150430E, NZ150526C, NK150527C, and CC151015C in Table 2). Queens were dissected in phosphate-buffered saline (PBS), and their spermathecae were removed under a stereomicroscope (SZX7; Olympus). Whole spermathecae of individual queens were immersed in fixation solution (4% paraformaldehyde) for 30 min at room temperature, washed with PBST (PBS with 0.1% Triton X-100) three times (5 min each), transferred into staining solution (propidium iodide [Dojindo, Kumamoto, Japan], final concentration: 2 ng/ml in PBST), and incubated for 20 min at room temperature. The spermathecae were washed with PBST for 10 min, mounted on glass slides with mounting medium (Vectashield H-1000; Vector Laboratories, Burlingame, CA), and kept at 4 °C until analysis. To acquire fluorescent images of spermathecae, we photographed the stained spermathecae using a confocal laser scanning microscope (TCS-SP8; Leica, Wetzlar, Germany) equipped with a 100×/1.4 oil HC PL APO objective. The parameter settings of the microscope were as follows: excitation wavelength, 561 nm; laser intensity, 20–40%; gain, 20–50; and laser type, DPSS 20 mW 561 nm. To compare the rate of insemination of egg-laying queens between asexual and sexual populations, we used Fisher’s exact probability test (Statistica 10; StatSoft, Tulsa, OK).

### Comparison of hatching success of unfertilized eggs between the asexual and sexual lineages

To investigate the difference in costs of parthenogenesis between the asexual and sexual lineages, we compared the hatching success of unfertilized eggs between the two lineages. Virgin alates were obtained from three stock colonies of the asexual lineage (AS141110A, AS141111I, and AS141111K in Table 1) and of the sexual lineage (IZ150430C, SN150430F, and SN150501A in Table 2), separated by sex before swarming began, and maintained in 90-mm Petri dishes containing moist unwoven clothes until they shed their wings. Then, two females, or a female and a male were randomly chosen from each colony and placed in a 52 × 76-mm glass cell that contained mixed sawdust bait blocks, as described in a previous study [41]. All treatments were replicated 20 times for each colony. The glass-cell colonies were kept at 25 °C under constant darkness for 100 days. We counted eggs and larvae by checking the glass-cell colonies every 3 days. The hatching success, calculated as percentage of eggs hatched within 100 days after colony foundation, was compared among eggs of glass-cell colonies founded by pairs of female reproductives (i.e., unfertilized eggs) of the asexual lineage, those of glass-cell colonies founded by pairs of female reproductives (i.e., unfertilized eggs) of the sexual lineage, and those of glass-cell colonies founded by pairs of female and male reproductives (i.e., fertilized eggs) of the sexual lineage using Fisher’s exact probability tests with Bonferroni correction (Statistica 10; StatSoft). Because egg protection behavior by reproductives is indispensable for egg survival, data for the glass-cell colonies in which at least one reproductive died were excluded from the analysis. Moreover, because there were no significant differences between the natal colonies and between the glass-cell colonies within egg types (unfertilized eggs of the asexual lineage, unfertilized eggs of the sexual lineage, and fertilized eggs of the sexual lineage), respectively (*P* > 0.05, Fisher’s exact probability test with Bonferroni correction [Statistica 10; StatSoft]), we pooled the data for both the natal colonies and the glass-cell colonies of each egg type.

### Phylogenetic analyses

To infer intraspecific relationships among asexual and sexual populations of *G*. *nakajimai*, we conducted phylogenetic analyses of *Glyptotermes* termites. One worker randomly chosen from each of the 15 *G*. *nakajimai* collection sites (six collection sites of asexual populations and nine collection sites of sexual populations) was used for phylogenetic analyses. The sequences of other kalotermitid species obtained in this study and from GenBank were also used in the analyses. Heads of individual termites were ground in Chelex-100 resin solution (Bio-Rad, Richmond, CA) and DNA was extracted and purified in accordance with standard Chelex-based protocols [52]. Individuals were sequenced for the mitochondrial cytochrome *c* oxidase subunit II (*COII*) and the nuclear internal transcribed spacer 2 (*ITS2*) genes. Primer sequences, PCR conditions, and sequencing methods are detailed in previous reports (*COII*: [53]; *ITS2*: [54]). We obtained *COII* (624 bp) and *ITS2* (444-bp) sequences. Consensus sequences were aligned using the ClustalX algorithm [55] from the BioEdit 7.0.4.1 sequence editor [56] and corrected manually. Bayesian inference was performed using MrBayes 3.1.2 [57] with the GTR + I + G model for *COII* and with the HKY + G model for *ITS2* selected by the hierarchical likelihood ratio test (hLRT) in MrModeltest 2.3 [58]. *Kalotermes flavicollis* (Fabricius) (GenBank accession number KX688868) and *G*. *fuscus* Oshima (GenBank accession number KX688884) were used as outgroups for the phylogenetic analyses of *COII* and *ITS2*, respectively. Methods of phylogenetic analyses are described in a previous report [53].

We also analyzed the sequence alignments using maximum likelihood in RAxML version 7.7.1 [59]. The GTRGAMMA model was selected for the combined datasets and 100 bootstrap replicates were performed. *Kalotermes flavicollis* (GenBank accession number KX688868) and *G*. *fuscus* (GenBank accession number KX688884) were used as outgroups for the phylogenetic analyses of *COII* and *ITS2*, respectively.

The *COII* and *ITS2* gene sequences obtained in this study were deposited in the DDBJ/EMBL/GenBank nucleotide sequence databases under accession numbers KX688845–KX688885.

### Divergence-dating analysis

We analyzed the *COII* DNA sequence alignment with a relaxed molecular-clock model using the Bayesian phylogenetic software BEAST 1.8.2. Rate variation was modeled among branches using uncorrelated lognormal relaxed clocks [60], with a single model for all genes. A Yule speciation process was used for the tree prior [61], and posterior distributions of parameters, including the tree, were estimated using MCMC sampling. We performed two replicate MCMC runs, with the tree and parameter values sampled every 1000 steps over a total of 50 million generations. A maximum clade credibility tree was obtained using Tree Annotator within the BEAST software package with a burn-in of 10,000 trees. Acceptable sample sizes and convergence to the stationary distribution were checked using Tracer 1.5 [60]. The molecular clock was calibrated using four minimum age constraints based on the following fossils: *Coptotermes priscus* Emerson, ~ 26 million years old; *Dolichorhinotermes dominicanus* Schlemmermeyer and Cancello, ~ 18 million years old; *Anoplotermes* sp., 18 million years old; and *Reticulitermes antiquus* (Germar), ~ 44 million years old; (for further details, see [62]). Fossil calibrations were implemented as exponential priors on node times.

### Cytological analysis

To examine the karyotypes of the asexual and sexual lineages, we used female alates of six stock colonies of the asexual lineage (TO150911B, MR150910B, AS141110A, SK150715A, TI150728A, and ST160304B in Table 1) and four stock colonies of the sexual lineage (IZ150430C, NK150527C, HD160328C, and HH151016F in Table 2), and male neotenic reproductives of two stock colonies of the sexual lineage (NK150527C and HH151016F in Table 2). Three alates or five neotenic reproductives were randomly chosen from each colony. The somatic chromosomes of alates and neotenic reproductives were observed using the lactic acid dissociation drying method described in previous studies [41, 63].

### Genome size analysis

To investigate the difference in genome size between the asexual and sexual lineages, we used female workers of three mature field colonies of the asexual lineage (TO150911A, MR150910A, and ST160304B in Table 1) and of the sexual lineage (IZ150430A, NZ150526C, and HH151016D in Table 2). Using flow cytometry with propidium iodide staining, we estimated genome size (*C* value). Tissue for flow cytometric analysis was processed with a Cycletest PLUS DNA Reagent Kit (Becton Dickinson, San Jose, CA). All procedures were performed in accordance with the manufacturer’s supplied protocol. Five female workers were randomly chosen from each colony and their heads were ground together with the heads of female *Drosophila melanogaster* Meigen (strain Oregon R) (1C = 0.18 pg [64]), which served as an internal standard, with a tight-fitting pestle. Detailed descriptions of the nuclear isolation and staining are described in a previous report [65]. Stained nuclei were analyzed for DNA-PI fluorescence using an Accuri C6 Flow Cytometer (Becton Dickinson) at an excitation wavelength of 488 nm. Approximately 1000 cells were acquired for each measurement, and gating was performed using Accuri C6 software v1.0.264.21 (Becton Dickinson). Each *C* value (pg) was calculated based on the main peak of 2C cells (G_0_/G_1_ phase) of *G*. *nakajimai* and *D*. *melanogaster*. To compare the genome size (*C* value) between the asexual and sexual lineages, we used nested ANOVA. Assuming that the sexual lineage is diploid, as are all known termite species [11], a relative ploidy level was assigned to the asexual lineage using the following formula: ploidy level = mean genome size of the asexual lineage/mean genome size of the sexual lineage × 2.

### Examination of sex differences in soldier morphology

To compare morphology between male and female soldiers, we used soldiers of five mature field colonies of the sexual lineage (SN150501A, CC151015B, HH151016D, HH151016F, and HH151016H in Table 2). Head width and head length were measured for all collected male and female soldiers, excluding severely damaged individuals, from each colony. Head width, head length, the head width to length ratio (head width/head length) were analyzed using two-way ANOVA (Statistica 10; StatSoft, Tulsa, OK).

### Comparison of the within-colony CV of soldier head width between the asexual and sexual lineages

To investigate whether asexuality leads to stabilization of soldier head width, we compared the within-colony coefficient of variation (CV = standard deviation/mean) of soldier head width between the asexual and sexual lineages. Excluding severely damaged individuals, all collected soldiers from five mature field colonies of the asexual lineage (TO150911A, MR150217B, MR150910B, AS141110A, and AS141111I in Table 1) and of the sexual lineage (SN150501A, CC151015B, HH151016D, HH151016F, and HH151016H in Table 2) were used for comparison. Head width was measured under a stereoscope (SZX7; Olympus) using a digital imaging system (FLVFS-LS; Flovel, Tokyo, Japan). Within-colony CVs of soldier head width, adjusted for sample size differences [66], were compared between the asexual and sexual lineages using Mann–Whitney *U* test (Statistica 10; StatSoft).

### Development of microsatellite markers

To characterize the asexual and sexual lineages of *G*. *nakajimai* genetically, we developed six polymorphic microsatellite markers for termites of the genus *Glyptotermes*. Genomic DNA was extracted from a pool of ten individuals from a single colony of *G*. *fuscus* collected on Okinawa Island using the DNeasy tissue Kit (Qiagen, Hilden, Germany). Microsatellite enrichment was achieved using streptavidin-coated magnetic spheres according to the protocol described in a previous study [49]. Enrichment amplicons were cloned using a pGEM T-Easy cloning kit (Promega, Madison, WI) following the manufacturer’s instructions. Inserted DNA obtained from the color-positive clones was amplified by PCR. PCR products were checked for the insert size by agarose gel electrophoresis with a 100-bp ladder, and PCR products exhibiting a single band of 400–1000 bp were sequenced. Primer sequences, PCR conditions, and sequencing methods are detailed in a previous report [49].

Primers were designed for the 18 inserts containing a microsatellite region with more than nine repeat units. Genomic DNA was extracted from each head of 15 individuals per colonies of *G*. *fuscus* collected on Okinawa Island, those of the sexual lineage of *G*. *nakajimai* (two to three colonies for each population: IZ150430A, IZ150430B, SN150430F, SN150501A, NZ150526A, NZ150526F, NK150527C, HD160328B, HD160328C, NJ150226A, YJ150226B, CC151014G, CC151015B, HH151016D, and HH151016E in Table 2) and those of the asexual lineage of *G*. *nakajimai* (three to four colonies for each population: TO150911A, TO150911B, MR150217B, MR150910B, MR150910D, AS141110A, AS141111H, AS141111K, SK150715A, SK150715B, TI150324A, TI150728A, ST160304A, ST160304B, and ST160304C in Table 1). PCR was carried out in accordance with the following protocol. Each of the 25 μL reaction mixtures contained 0.5 μM each primer (for detailed information on the primers, see Additional file 4: Table S1), 1× PCR buffer, 0.5× Q-solution, 0.5 mM MgCl_2_, 0.25 mM each dNTP, 0.75 U Taq DNA polymerase (Qiagen), and 1 μL of template DNA. The reactions were run according to the PCR cycle, which consisted of an initial denaturation step at 94 °C for 3 min, followed by 35 cycles of 30 s at 94 °C and 60 s at 60 °C, and one step at 72 °C for 2 min to complete the extension at the end. The PCR products were electrophoresed with the GeneScan-600 LIZ size standard (Applied Biosystems, Foster City, CA) on a 3500 Genetic Analyzer (Applied Biosystems).

Of 18 loci, polymorphisms within the Okinawa Island population of *G*. *fuscus* were found for six (Additional file 5: Table S2). Genetic diversity based on number of alleles, observed, and expected heterozygosities, and deviations from Hardy–Weinberg equilibrium for each locus in the Okinawa Island population of *G*. *fuscus*, the sexual and asexual lineages of *G*. *nakajimai*, respectively, were calculated using Genepop on the Web 4.2 [67]. When appropriate, Bonferroni correction for multiple tests (Statistica 10; StatSoft) was applied.

### Mode of parthenogenesis in *G*. *nakajimai*

To assess the mode of parthenogenesis in the sexual lineage of *G*. *nakajimai*, we genotyped the primary queens and larvae in the glass-cell colonies founded by pairs of female reproductives (the asexual laboratory colonies) described above. All individuals used in this analysis were placed in vials containing 99.5% (vol/vol) ethanol and stored until DNA extraction. Heads of queens or whole larvae were ground in Chelex-100 resin solution (Bio-Rad, Richmond, CA), and DNA was extracted and purified in accordance with standard Chelex-based protocols [52]. Individuals were genotyped at two polymorphic microsatellite loci: *Gly8* and *Gly18* (Additional file 4: Table S1 and Additional file 5: Table S2). PCR and electrophoresis were performed as described above.

## Additional files

Additional file 1: **Figure S1.** Sexual dimorphism in the external morphology of *Glyptotermes nakajimai*. Ventral view (posterior-up) of the caudal sternites and dorsal view (anterior-up) of the gonads of male (left) and female (right) reproductives (top), soldiers (middle), and workers (bottom). 7th, seventh sternite; T, testes; OV, ovaries. Scale bars, 400 μm. (PDF 3880 kb)

Additional file 2: **Figure S2.** Maximum likelihood trees of mitochondrial *COII* (left) and nuclear *ITS2* (right) sequences of *Glyptotermes nakajimai* individuals representing each of the collection sites. The asexual lineage is highlighted in red, and the sexual lineage is highlighted in blue. Bootstrap values > 50% are shown by the branch. The horizontal bar represents a distance of 0.1 substitutions per site. GenBank accession numbers are shown in parentheses. (PDF 427 kb)

Additional file 3: **Figure S3.** Differences in the head width (left), the head length (middle), and the head width to length ratio (right) between female (*n* = 99) and male (*n* = 102) soldiers of the sexual lineage. Parameters of the box-and-whisker plots: line, median; box, first to third quartile; upper whisker, third quartile + 1.5 × interquartile range; lower whisker = first quartile − 1.5 × interquartile range; black dots, outliers. **, *P* < 0.01; ***, *P* < 0.001 (two-way ANOVA). For raw data, see Additional file 7. (PDF 274 kb)

Additional file 4: **Table S1.** Newly developed microsatellite markers for termites of the genus *Glyptotermes*. (DOC 40 kb)

Additional file 5: **Table S2.** Characterization of six microsatellite loci in the asexual, sexual lineages of *Glyptotermes nakajimai*, and the related species *G*. *fuscus*. (DOC 51 kb)

Additional file 6: **Table S3.** Genotypes of the primary queens (PQ) and larvae (L) in the asexual laboratory colonies of the sexual lineage of *Glyptotermes nakajimai*. (DOC 45 kb)

Additional file 7: Supporting data. Raw data for Fig. 7b, c, Table 3, and Additional file 3: Figure S3. (XLSX 31 kb)
