# Supplementary figures and images for: Loss of males from mixed-sex societies in termites
Source: BMC Biol. 2018 Sep 25;16:96. doi: 10.1186/s12915-018-0563-y (PMC6154949; doi:10.1186/s12915-018-0563-y)

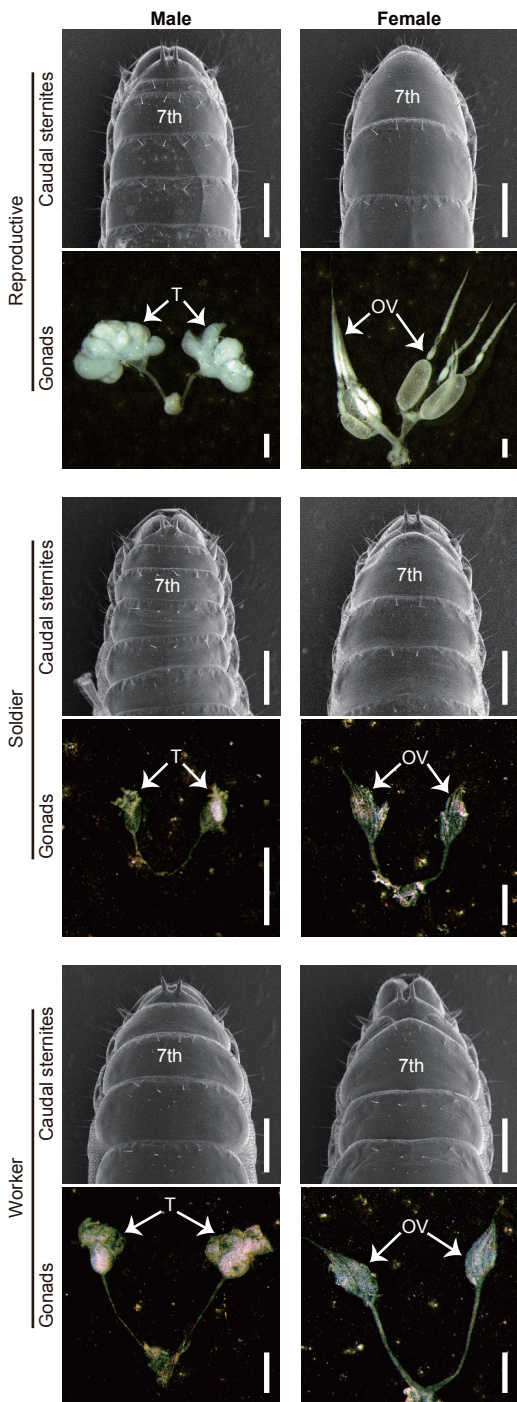

Supplement: Supplementary file 1 — Figure S1. Sexual dimorphism in the external morphology of Glyptotermes nakajimai. Ventral view (posterior-up) of the caudal sternites and dorsal view (anterior-up) of the gonads of male (left) and female (right) reproductives (top), soldiers (middle), and workers (bottom). 7th, seventh sternite; T, testes; OV, ovaries. Scale bars, 400 μm. (PDF 3880 kb) [file 12915_2018_563_MOESM1_ESM.pdf]

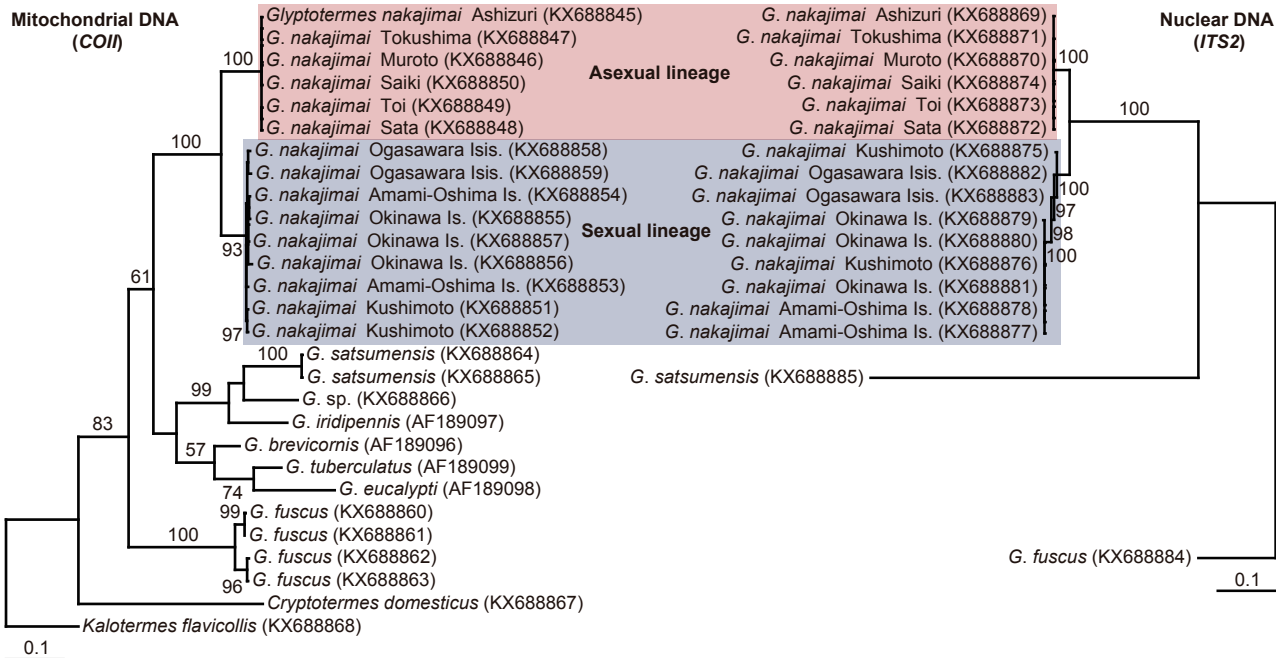

Supplement: Supplementary file 2 — Figure S2. Maximum likelihood trees of mitochondrial COII (left) and nuclear ITS2 (right) sequences of Glyptotermes nakajimai individuals representing each of the collection sites. The asexual lineage is highlighted in red, and the sexual lineage is highlighted in blue. Bootstrap values > 50% are shown by the branch. The horizontal bar represents a distance of 0.1 substitutions per site. GenBank accession numbers are shown in parentheses. (PDF 427 kb) [file 12915_2018_563_MOESM2_ESM.pdf]

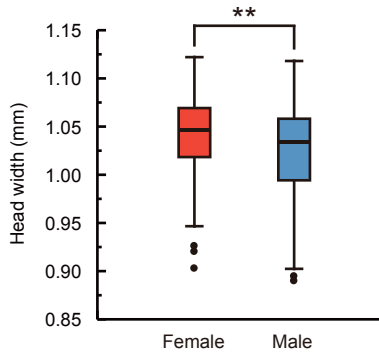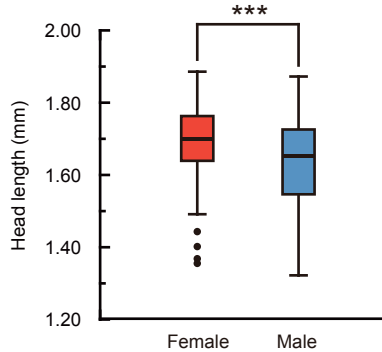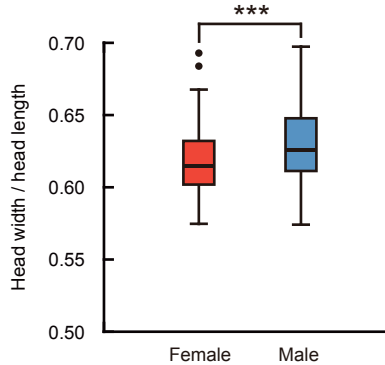

Supplement: Supplementary file 3 — Figure S3. Differences in the head width (left), the head length (middle), and the head width to length ratio (right) between female (n = 99) and male (n = 102) soldiers of the sexual lineage. Parameters of the box-and-whisker plots: line, median; box, first to third quartile; upper whisker, third quartile + 1.5 × interquartile range; lower whisker = first quartile − 1.5 × interquartile range; black dots, outliers. **, P < 0.01; ***, P < 0.001 (two-way ANOVA). For raw data, see Additional file 7. (PDF 274 kb) [file 12915_2018_563_MOESM3_ESM.pdf]
