# Supplementary material for: Loss of males from mixed-sex societies in termites
Source: BMC Biol. 2018 Sep 25;16:96. doi: 10.1186/s12915-018-0563-y (PMC6154949; doi:10.1186/s12915-018-0563-y)
Supplement: Supplementary file 4 — Table S1. Newly developed microsatellite markers for termites of the genus Glyptotermes. (DOC 40 kb) [file 12915_2018_563_MOESM4_ESM.doc]

**Table S1** Newly developed microsatellite markers for termites of the genus *Glyptotermes*

| Locus | Primer sequences (5'–3') | Repeat motif | Size (bp) | GenBank no. |
| --- | --- | --- | --- | --- |
| *Gly01* | F: GCTGCTGGCAACGGTATGAA | (AC)_11_(GCAC)_10_(AC)_32_ | 252 | KY510435 |
|  | R: AGCGTGTACCTGGTCGCACA |  |  |  |
| *Gly02* | F: GGGTCTCGCCTGCTGAATGT | (AC)_9_ | 188 | KY510436 |
|  | R: GGTTTCATGCATCGGCAGTG |  |  |  |
| *Gly04* | F: AACGAAATGGACGTGCAGGA | (GT)_15_ | 277 | KY510437 |
|  | R: CGATGTTTGCGGACGAACAG |  |  |  |
| *Gly08* | F: CTGCTGGCGCGTAAGTCTGA | (GT)_11_ | 360 | KY510438 |
|  | R: TGCCCAGAGCTCATGTATGGA |  |  |  |
| *Gly10* | F: CAATGGCAAACTTCAGGAATGG | (CT)_9_ | 159 | KY510439 |
|  | R: GCAGACGTGTCAGCATGCACT |  |  |  |
| *Gly18* | F: GCAACCGCTCACCAAAACCT | (CT)_10_ | 368 | KY510440 |
|  | R: TTGGTAGCGCCGTCGTCATA |  |  |  |

F, forward; R, reverse
