# Supplementary material for: Loss of males from mixed-sex societies in termites
Source: BMC Biol. 2018 Sep 25;16:96. doi: 10.1186/s12915-018-0563-y (PMC6154949; doi:10.1186/s12915-018-0563-y)
Supplement: Supplementary file 5 — Table S2. Characterization of six microsatellite loci in the asexual, sexual lineages of Glyptotermes nakajimai, and the related species G. fuscus. (DOC 51 kb) [file 12915_2018_563_MOESM5_ESM.doc]

**Table S2** Characterization of six microsatellite loci in the asexual, sexual lineages of *Glyptotermes* *nakajimai*, and the related species *G*. *fuscus*

| Locus | *N*_A_ | Size range (bp) | *H*_O_ | *H*_E_ | *P* |
| --- | --- | --- | --- | --- | --- |
| Asexual lineage of *G*. *nakajimai* |  |  |  |  |  |
| *Gly01* | 1 | 252 | – | – |  |
| *Gly02* | 1 | 232 | – | – |  |
| *Gly04* | 1 | 251 | – | – |  |
| *Gly08* | 1 | 314 | – | – |  |
| *Gly10* | 1 | 131 | – | – |  |
| *Gly18* | 1 | 422 | – | – |  |
| Sexual lineage of *G*. *nakajimai* |  |  |  |  |  |
| *Gly01* | 1 | 252 | – | – |  |
| *Gly02* | 1 | 232 | – | – |  |
| *Gly04* | 1 | 251 | – | – |  |
| *Gly08* | 2 | 314-326 | 0.267 | 0.460 | 0.232 |
| *Gly10* | 1 | 131 | – | – |  |
| *Gly18* | 2 | 420-422 | 0.133 | 0.239 | 0.199 |
| *G*. *fuscus* |  |  |  |  |  |
| *Gly01* | 2 | 252-254 | 0.000 | 0.129 | 0.033* |
| *Gly02* | 2 | 186-188 | 0.000 | 0.129 | 0.034* |
| *Gly04* | 5 | 277-287 | 0.400 | 0.584 | 0.288 |
| *Gly08* | 4 | 356-364 | 0.533 | 0.680 | 0.232 |
| *Gly10* | 2 | 159-161 | 0.467 | 0.480 | 1.000 |
| *Gly18* | 4 | 358-372 | 0.200 | 0.306 | 0.077 |

*N*_A_, number of alleles; *H*_O_, observed heterozygosity; *H*_E_, expected heterozygosity; *P*, *p*-value associated with departure from Hardy–Weinberg Equilibrium

**P* < 0.05, but not significant after Bonferroni correction
