# Supplementary material for: Loss of males from mixed-sex societies in termites
Source: BMC Biol. 2018 Sep 25;16:96. doi: 10.1186/s12915-018-0563-y (PMC6154949; doi:10.1186/s12915-018-0563-y)
Supplement: Supplementary file 6 — Table S3. Genotypes of the primary queens (PQ) and larvae (L) in the asexual laboratory colonies of the sexual lineage of Glyptotermes nakajimai. (DOC 45 kb) [file 12915_2018_563_MOESM6_ESM.doc]

**Table S3** Genotypes of the primary queens (PQ) and larvae (L) in the asexual laboratory colonies of the sexual lineage of *Glyptotermes* *nakajimai*

|  |  | Locus | |
| --- | --- | --- | --- |
| Colony^a^ | Individual | *Gly8*^b^ | *Gly18*^b^ |
| FF_IZ150430A_-8 | PQ-1^c^ | 326/326 | **420**/422 |
|  | PQ-2 | 326/326 | 422/422 |
|  | L-1 | 326/326 | **420**/**420** |
| FF_IZ150430A_-17 | PQ-1^c^ | 326/326 | **420**/422 |
|  | PQ-2 | 326/326 | 422/422 |
|  | L-1 | 326/326 | **420**/**420** |
| FF_IZ150430A_-18 | PQ-1 | 326/326 | 420/420 |
|  | PQ-2 | 326/326 | 420/420 |
|  | L-1 | 326/326 | 420/420 |
| FF_SN150430F_-8 | PQ-1^c^ | 314/**326** | 422/422 |
|  | PQ-2 | 314/314 | 420/422 |
|  | L-1 | **326**/**326** | 422/422 |
| FF_SN150430F_-19 | PQ-1^c^ | **326**/326 | 420/422 |
|  | PQ-2 | 314/314 | 420/422 |
|  | L-1 | **326**/**326** | 422/422 |
|  | L-2 | **326**/**326** | 422/422 |
| FF_SN150501A_-12 | PQ-1^c^ | 314/**326** | 422/422 |
|  | PQ-2 | 314/314 | 420/422 |
|  | L-1 | **326**/**326** | 422/422 |

Asexually produced offspring are homozygous for a single maternal allele at all two loci

^a^Subscripts in colony codes indicate natal colonies, shown in Table 2, of female founders (FF)

^b^Inferred mothers’ alleles inherited by asexually produced larvae are indicated in bold

^c^Inferred mothers of asexually produced larvae
